# An assessment of esophageal balloon use for the titration of airway pressure release ventilation and controlled mechanical ventilation in a patient with extrapulmonary acute respiratory distress syndrome: a case report

**DOI:** 10.1186/s13256-021-02984-2

**Published:** 2021-08-17

**Authors:** Óscar Arellano-Pérez, Felipe Castillo Merino, Roberto Torres-Tejeiro, Sebastián Ugarte Ubiergo

**Affiliations:** 1 Adult Patients Critical Center, INDISA Clinic, Santiago, Chile; 2 School of Physical Therapy, Faculty of Rehabilitation Sciences, Andrés Bello University, Santiago, Chile; 3 School of Physical Therapy, Faculty of Health, Bernardo O’Higgins University, Santiago, Chile; 4 Faculty of Medicine, Andrés Bello University, Santiago, Chile; 5 Latin American Critical Care Trial Investigative Network (LACCTIN), Santiago, Chile

**Keywords:** Mechanical ventilation, ARDS, Transpulmonary pressure, Esophageal balloon

## Abstract

**Background:**

Esophageal pressure measurement is a minimally invasive monitoring process that assesses respiratory mechanics in patients with acute respiratory distress syndrome. Airway pressure release ventilation is a relatively new positive pressure ventilation modality, characterized by a series of advantages in patients with acute respiratory distress syndrome.

**Case presentation:**

We report a case of a 55-year-old chilean female, with preexisting hypertension and recurrent renal colic who entered the cardiosurgical intensive care unit with signs and symptoms of urinary sepsis secondary to a right-sided obstructive urolithiasis. At the time of admission, the patient showed signs of urinary sepsis, a poor overall condition, hemodynamic instability, tachycardia, hypotension, and needed vasoactive drugs. Initially the patient was treated with volume control ventilation. Then, ventilation was with conventional ventilation parameters described by the Acute Respiratory Distress Syndrome Network. However, hemodynamic complications led to reduced airway pressure. Later she presented intraabdominal hypertension that compromised the oxygen supply and her ventilation management. Considering these records, an esophageal manometry was used to measure distending lung pressure, that is, transpulmonary pressure, to protect lungs. Initial use of the esophageal balloon was in a volume-controlled modality (deep sedation), which allowed the medical team to perform inspiratory and expiratory pause maneuvers to monitor transpulmonary plateau pressure as a substitute for pulmonary distension and expiratory pause and determine transpulmonary positive end-expiratory pressure. On the third day of mechanical respiration, the modality was switched to airway pressure release ventilation. The use of airway pressure release ventilation was associated with reduced hemodynamic complications and kept transpulmonary pressure between 0 and 20 cmH_2_O despite a sustained high positive end-expiratory pressure of 20 cmH_2_O.

**Conclusion:**

The application of this technique is shown in airway pressure release ventilation with spontaneous ventilation, which is then compared with a controlled modality that requires a lesser number of sedative doses and vasoactive drugs, without altering the criteria for lung protection as guided by esophageal manometry.

## Introduction

Esophageal pressure (Pes) measurement is a minimally invasive monitoring method used to assess respiratory mechanics in acute respiratory distress syndrome [1]. Pes tracings can be used to understand, define, and individually assess the physiopathological mechanisms of respiratory insufficiency and monitor the patient’s clinical progression [1]. The first generation of customized esophageal balloons was mainly used for research purposes. Over the past decade, several kinds of second-generation balloons have been developed and are currently available for clinical use [1]. Esophageal balloon catheters can be connected to specific monitoring devices, such as pressure ports, mechanical ventilator accessories, or multiparametric monitor pressure transductors [1]. This kind of additional information can be useful in the case of pathologies such as acute respiratory distress syndrome (ARDS), which is characterized by acute respiratory insufficiency with bilateral shadowing on thorax X-rays, pulmonary edema that cannot be completely explained by cardiac insufficiency or excess lung liquid, and hypoxemia with a PaO_2_/FiO_2_ ratio < 300 with positive end-expiratory pressure (PEEP) higher or equal to 5 cmH_2_O [2]. Despite the usefulness of esophageal pressure monitoring, clinical reports are still scarce, and its use in unconventional modalities is experimental and mainly used in research. To better clarify the usefulness of Pes measurements in a clinical context, we will assess the technical, physiological, and clinically relevant details of this monitoring method to facilitate an improved understanding of the information provided by bedside Pes measurements.

Airway pressure release ventilation (APRV) was first described and introduced to clinical practice over 20 years ago and was made commercially available by the mid 1990s [3, 4]. It is a relatively new positive pressure ventilation modality and has a series of advantages over low tidal volume assisted ventilation in ARDS patients [5]. Its benefits are mainly related to spontaneous respiration, which improves both patient–ventilator synchronization and the ventilation-to-perfusion ratio, thus improving gas distribution into dependent lung regions [6].

## Case study

**Fig. 1:**
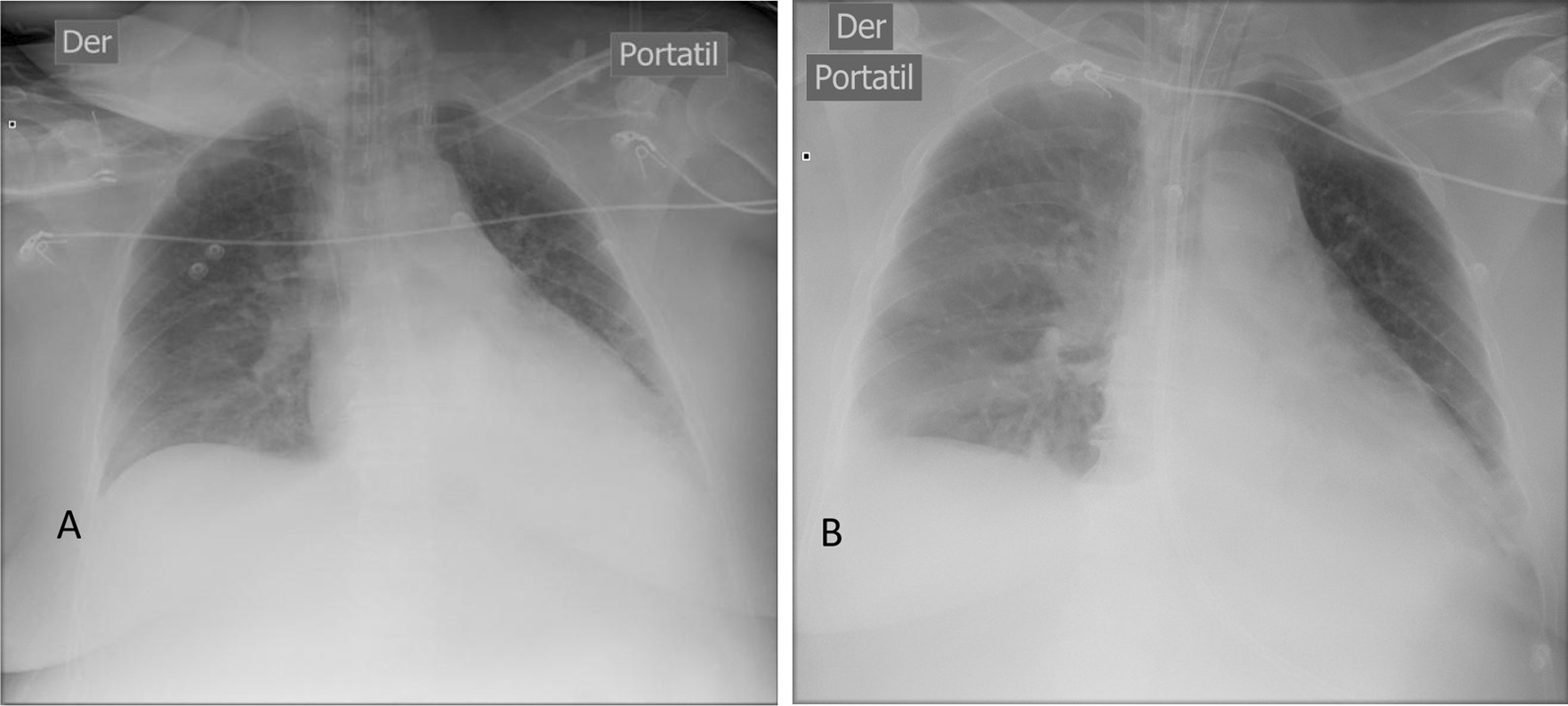
**A** Thoracic X-ray on first day in intensive care unit / **B** Thoracic X-ray on third day in intensive care unit

The patient was a 55-year-old chilean female, with preexisting hypertension and recurrent renal colic who entered the cardiosurgical intensive care unit (ICU) with signs and symptoms of urinary sepsis secondary to a right-sided obstructive urolithiasis. Upon admission, the patient showed signs of urinary sepsis, a poor overall condition, hemodynamic instability, tachycardia, hypotension mean arterial pressure (MAP) 70, and required vasoactive drugs. A general physical examination showed signs of consciousness and a Richmond Agitation–Sedation Scale (RASS) score of −1 to 0. The patient had petechiae on her upper torso and lower limbs; an apparently painless globular, soft, and depressible abdomen; a medial laparotomy scar; and a left lumbotomy scar. The APACHE II (first 24 hours) score was 19 (Table 1; Fig. 1).

**Table 1:** Patient description

Age	55 years old
Sex	Female
Weight	76 kg
Height	162 cm
Body mass index	29
APACHE II (score)	19
ICU stay (days)	14
Days under invasive mechanical ventilation	7
Days under invasive mechanical ventilation with esophageal balloon	5
Days under deep sedation at ICU	6
Day consciousness was regained	6
First day of sitting on the edge of the bed	7
First day standing	7
Scale for muscle strength (Medical Research Council Sum Score) regaining consciousness	30

## Initial ventilation management

The patient was treated with volume control ventilation. A lung recruitment maneuver was performed following a first arterial blood gas test (ABGs) (Table 2, Day 0, first rounds), as oxygenation following the intervention was inadequate (Table 2, Day 1, first rounds), with the highest PEEP being 20 cmH_2_O. Until day 2, ventilation was volume controlled with conventional ventilation parameters described by the Acute Respiratory Distress Syndrome Network (ARDSNet) [2]. However, pressure on the airway was reduced owing to hemodynamic complications (Table 2, day 1 first round to day 2 fourth round).

**Table 2:** Respiratory therapy register—conventional mechanical ventilation without esophageal balloon

Day / round number	Day 0	Day 1 / 1	Day 1 / 2	Day 1 / 3	Day 1 / 4	Day 1 / 5	Day 2 / 1	Day 2 / 2	Day 2 / 3	Day 2 / 4
Mechanical ventilator modality	AC/VC	AC/VC	AC/VC	AC/VC	AC/VC	AC/VC	AC/VC	AC/VC	AC/VC	AC/VC
Programmed respiratory frequency (breaths per minute)	28	28	24	20	20	22	22	22	22	22
Total respiratory frequency (breaths per minute)	29	28	24	20	20	22	22	22	22	22
Programmed tidal volume (ml)	370	370	370	420	420	370	370	370	370	370
Expiratory tidal volume (ml)	360	392	386	420	430	376	376	377	388	385
Expiratory minute volume (ml)	11.9	10.9	9.23	8.57	8.58	8.25	8.27	8.28	8.47	8.53
Peak inspiratory flow rate (liters per minute)	38	48	48	48	48	34	34	34	34	34
Inspiratory time (seconds)	1.06	0.84	0.84	0.92	0.92	1.18	1.18	1.18	1.19	1.19
Expiratory time (seconds)	1.08	1.3	1.66	2.08	2.08	1.55	1.55	1.55	1.55	1.55
Maximum pressure (cmH_2_O)	27	32	33	30	32	26	26	30	34	31
Plateau pressure (cmH_2_O)	20	29	30	24	26	25	25	29	35	29
Mean airway pressure (cmH_2_O)	18	25	25	17	18	18	18	22	22	19
PEEP / PEEP Low (APRV) (cmH_2_O)	10	20	20	12	12	12	12	16	16	12
Intrinsic PEEP (cmH_2_O)	0.7	0.7	0.3	0.5	0.5	0.8	0.7	0.7	0.8	0.8
FiO_2_	100	90	50	60	60	60	60	60	60	90
SpO_2_	87	92	96	93	100	100	100	99	85	95
Respiratory system compliance (Static)	25	41	34	38	22	30	32	36	35	34
Static resistance	11	5.8	5.5	7.3	8.6	6.3	6.2	9.8	7.4	7.2
pH	7.238	7.412	–	7.41	7.42	7.534	7.529	7.52	7.41	7.45
PCO_2_ (mmHg)	49.7	31	–	30.1	31.7	33.6	29	31	38.8	37.3
PO_2_ (mmHg)	42	234	–	86.1	105.2	96.7	61	110	48.8	57
HCO_3_ (mmol/L)	20.7	19.3	–	19	20.2	27.7	24.2	24.8	24.1	25.6
B.E.	−6.9	−4.1	–	−4.1	−3.1	5.4	1	2.8	−0.3	1.9
Sat O_2_ (%)	68.6	99.5	–	96.8	98	98	94	98.5	85	91.1
PaO_2_/FiO_2_	42	260	–	143	175	161.1	101.6	183.3	81	131
Iox	42.8	9.6	–	11.8	10.2	11.1	17.7	12	27.1	14.5

AC/VC: Volume-controlled assisted modality; PEEP: Positive end-expiratory pressure; FiO_2_: Fraction of inspired oxygen; SpO_2_: Pulse oximetry saturation; PCO_2_: Arterial partial pressure of carbon dioxide; PO_2_: Arterial partial pressure of oxygen; HCO_3_: Bicarbonate; B.E: Base excess; SaO_2_: Arterial oxygen saturation; Iox: Oxygenation Index

## Transpulmonary pressure monitoring under controlled ventilation

During the patient’s second day on mechanical ventilation, she showed intraabdominal hypertension [intraabdominal pressure (IAP) 18 mmHg] that compromised the oxygen supply and her ventilation management (Table 2, day 2, second and third rounds). Consequently, an esophageal manometry was taken to measure lung distention [that is, transpulmonary pressure (TPP); 7] and protect the patient’s lungs. Initial use of the esophageal balloon volume-controlled in deep sedation allowed the medical team to perform inspiratory and expiratory pause maneuvers to monitor transpulmonary pressure during end inspiration (PLend-insp) and transpulmonary pressure during end expiration (PLend-exp) (Table 3, Day 2, fourth and fifth rounds). In line with the literature on esophageal manometry catheter use, our aim was to attain a PLend-insp below 20 cmH_2_O, as part of the lung enters full regional pulmonary capacity at this point [8].

**Table 3:** Respiratory therapy record (GSA programming and control)—conventional mechanical ventilation with esophageal balloon

Day / round number	Day 2 / 5	Day 3 /1	Day 3 / 2	Day 3 / 3	Day 3 / 4	Day 4 / 1	Day 4 / 2	Day 4 / 3	Day 4 / 4	Day 4 / 5	Day 4 / 6
Mechanical ventilator modality	AC/VC	AC/VC	AC/VC	AC/VC	APRV	APRV	APRV	APRV	APRV	AC/VC	AC/VC
Programmed respiratory frequency (revolutions per minute)	22	18	18	18	16	16	16	13	11	22	22
Total respiratory frequency (revolutions per minute)	22	18	19	18	22	21	20	25	25	25	23
Programmed tidal volume (ml)	370	440	440	440	–	–	–	–	–	320	320
Expiratory tidal volume (liters per minute)	360	440	420	435	360	440	470	520	500	350	355
Expiratory minute volume (liters per minute)	7.8	7.1	7.1	7.0	7.4	8.5	9.7	8.9	8.3	7.9	8.05
Inspiratory flux (liters per minute)	30	50	50	50	–	–	–	–	–	30	30
Expiratory time (seconds)	1.7	2.47	2.47	2.47	0.6	0.6	3.1	5	5	0.64	0.64
Peak pressure (cmH_2_O)	34	26	24	24	25	25	29	29	29	28	28
PEEP High (APRV) (cmH_2_O)	–	–	–	–	20	22	22	26	26	–	–
Plateau pressure (cmH_2_O)	35	31	28	28	–	–	–	–	–	22	21
Mean airway pressure (cmH_2_O)	25	20	19	19	21	22	22	26	26	21	21
PEEP / PEEP Low (APRV) (cmH_2_O)	20	16	15	15	10	10	12	12	12	18	18
Support pressure (cmH_2_O)	–	–	–	–	4	5	5	2	2	–	–
FiO2 (%)	100	100	90	80	80	80	70	80	80	80	60
SpO2 (%)	95	96	100			100	99	100	100	100	97
Respiratory system compliance (Static)		30	32	–	–	–	–	–	–	45	42
PLend-insp (cmH_2_O)	11	13	8			13	13	17	12	7	5
Plend-exp (cmH_2_O)	0	2	−2			−2	−2	−1	−1	1	2
pH	7.35	7.4	7.43	7.44	7.41	7.46	7.49	7.47	7.45	7.43	7.39
PCO_2_ (mmHg)	37.6	41.4	38.5	40.3	41.4	36.1	32.8	33.7	35	37.2	40.1
PO_2_ (mmHg)	69.9	96.7	94.6	92	82.3	103.3	63.6	90	86.1	141.5	87.4
HCO_3_ (mmol/L)	22.4	25.4	25.4	26.8	27.1	25.3	24.5	24.2	23.9	24.5	24.1
B.E.	−2.1	−0.6	1.3	2.6	1.2	1.8	1.9	1.2	0.5	0.6	−0.6
Sat O_2_ (%)	94	97.4	97.5	97.3	96.2	98	94.1	97.4	97	98.9	96.6
PaO_2_/FiO_2_	69.9	96.7	105.1	115	102	128	90	112	107	176	145
Iox	35.7	20.6	18.07	16.5	20.5	17.1	24.4	23?	24.2	11.9	14.4

AC/VC: Volume-controlled assisted modality; APRV: Airway pressure release ventilation; PEEP: Positive end-expiratory pressure; FiO_2_: Fraction of inspired oxygen; PLend-insp: Transpulmonary pressure during end inspiration; PLend-exp: Transpulmonary pressure during end expiration; SpO_2_: Pulse oximetry saturation; PCO_2_: Arterial partial pressure of carbon dioxide; PO_2_: Arterial partial pressure of oxygen HCO_3_: Bicarbonate; B.E: Base excess; SaO2: Arterial oxygen saturation; Iox: Oxygenation Index

## Catheter insertion with an esophageal balloon

The AVEA (Care Fusion) ventilator’s esophageal manometry system was used, following a test of the balloon and pressure measurement calibration. The catheter was then inserted through the nasal passage to approximately 55 cm, in line with the available literature [9], until reaching optimum gastric position, before being inflated to a suitable volume so measurements would remain unaltered. The catheter’s intragastric placement was checked by means of positive pressure deviation under gentle external manual epigastric compression [1]. The balloon was then removed until cardiac activity was visible in the esophageal pressure reading, indicating that the pressure measuring location was in the bottom third of the esophagus. Since the Pes measurement [7] did not seem to be significantly affected by the presence of a nasogastric tube, the patient was also given a nasojejunal probe. An occlusion test (Baydur test) [10] was performed to determine the accurate measurement of the esophageal balloon.

**Fig. 2:**
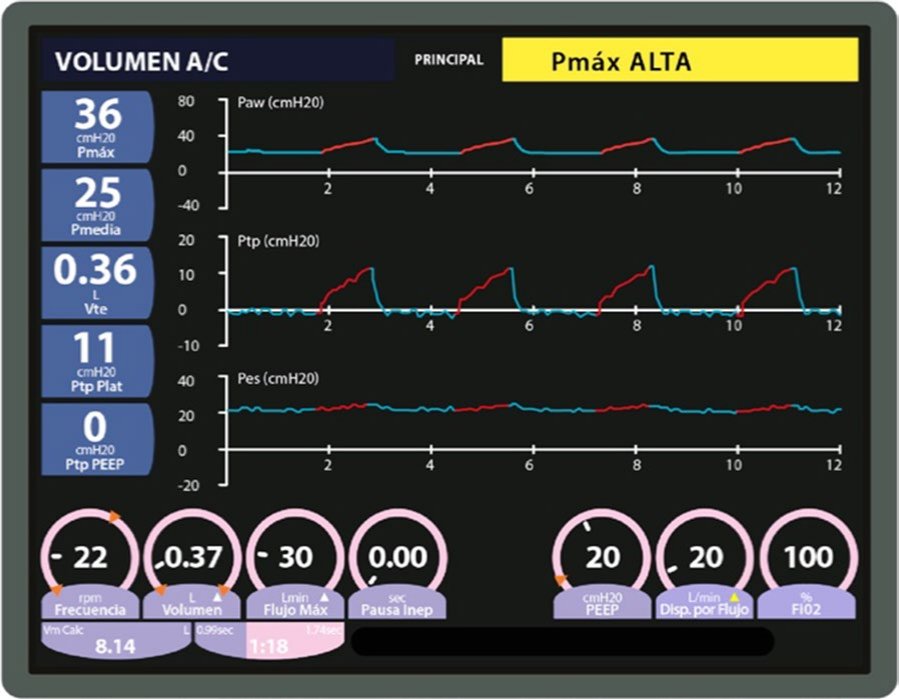
Mechanical ventilation programming on the first day the esophageal balloon was installed. Patient shows intraabdominal hypertension (18 mmHg IAP)

**Fig. 3:**
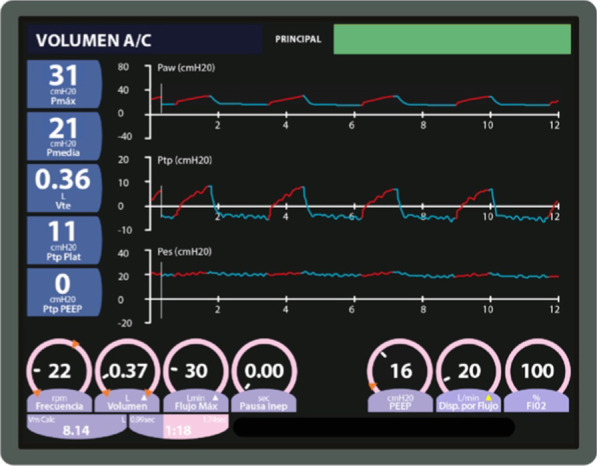
Mechanical ventilation programming during day 1 of esophageal balloon use. Patient presents intraabdominal hypertension with 18 mmHg IAP (previous 16 PEEP setting was insufficient)

A 20 cmH_2_O PEEP was initially established to maintain a 0 and 2 cmH_2_O PLend-exp (Fig. 2). However, hemodynamic deterioration made it necessary to establish a PEEP of up to 16 cmH_2_O, leading to a −2 and −1 PLend-exp (Figs. 3, 6A).

## Monitoring transpulmonary pressure in APRV

**Fig. 4:**
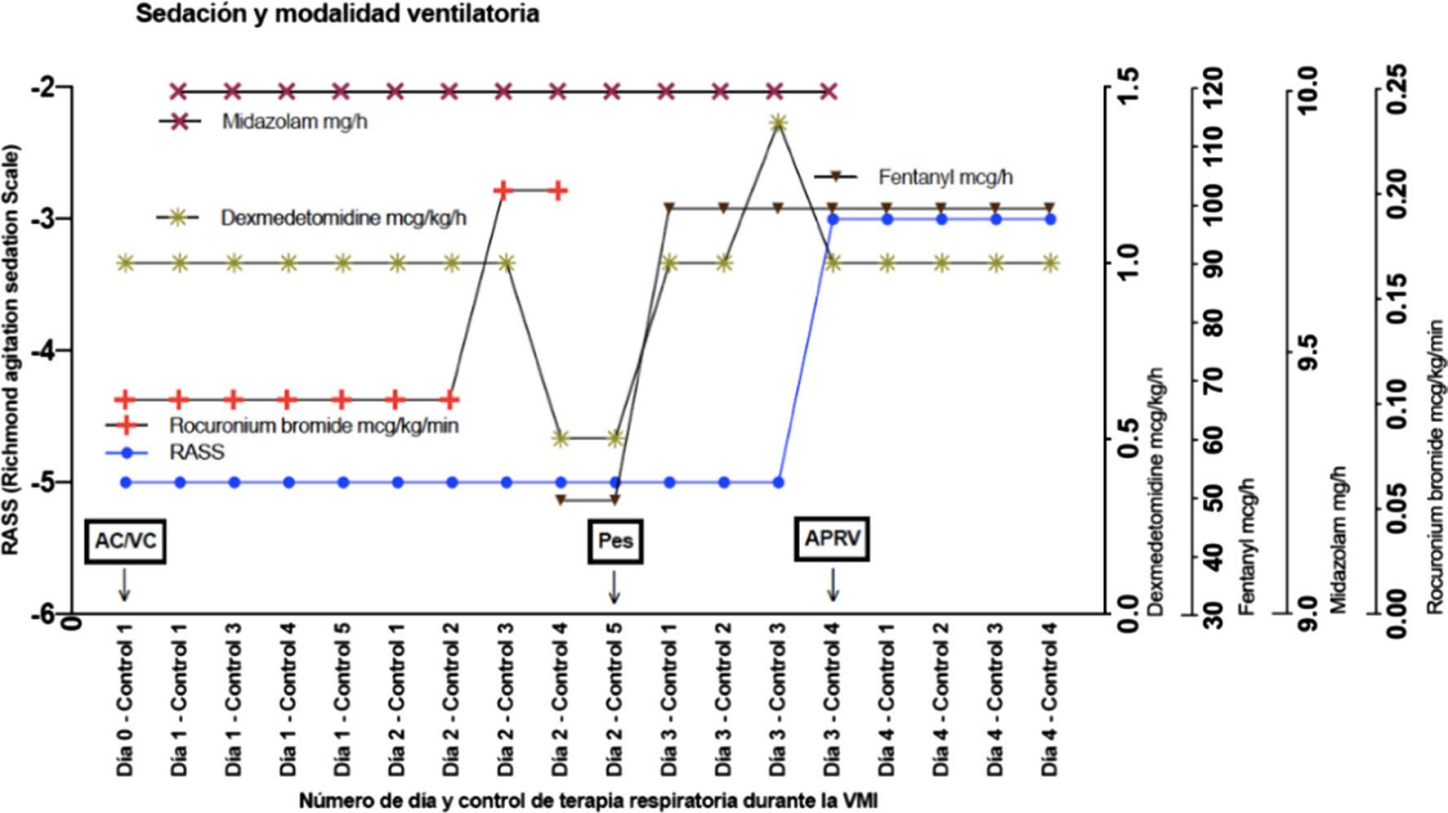
Sedation. Doses of dexmedetomidine, fentanyl, midazolam, and rocuronium bromide, and ventilation modality. *AC/VC* volume-controlled assisted modality, *AC/PC* pressure-controlled assisted modality, *APRV* airway positive pressure release ventilation, *Pes* esophageal pressure

On the third day of mechanical ventilation, the modality was switched to APRV (PEEP high of 20 cmH_2_O and low of 10 cmH_2_O, peak timing of 3.1 seconds, 0.6 seconds expiratory time, 16 revolutions per minute mandatory respiratory frequency, and 80% FiO_2_), which allowed for lower sedation levels, from –5 to −3 RASS, from the suspension of continuous midazolam infusion (Fig. 4).

**Fig. 5:**
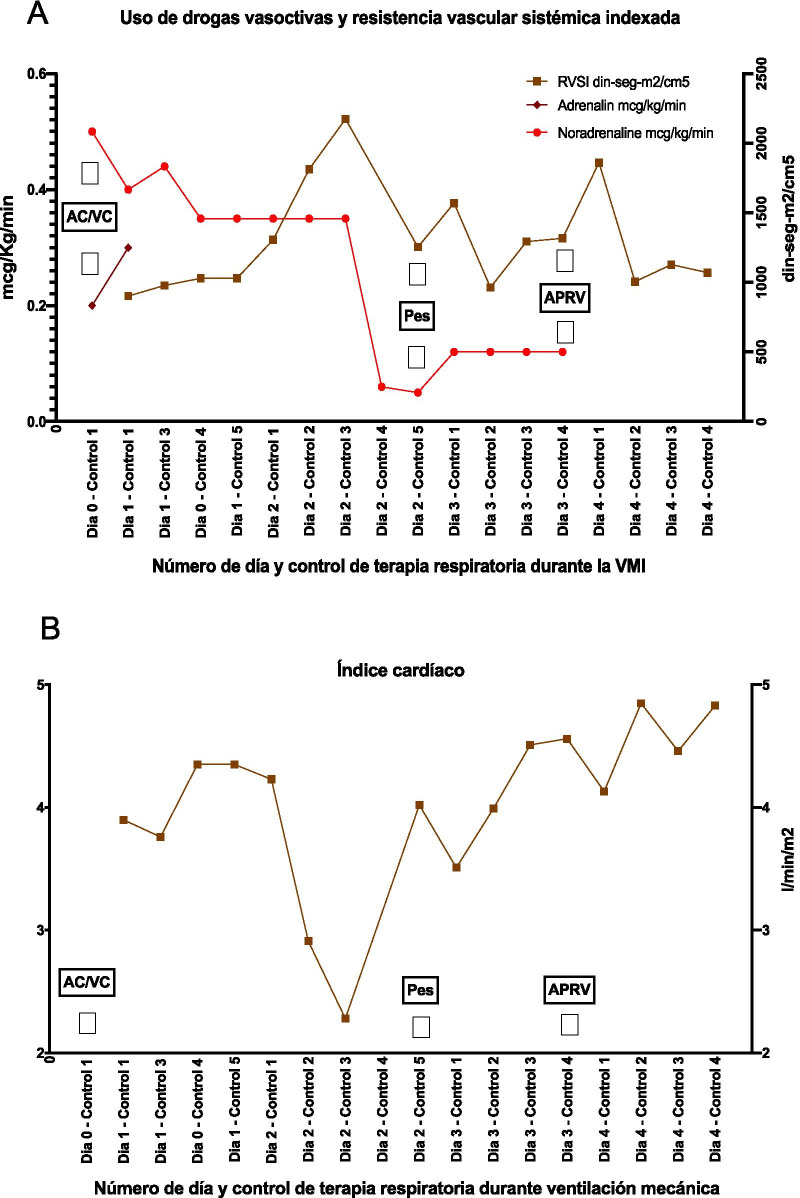
Hemodynamic monitoring via thermodilution (PiCCO System). **A** Vasoactive drug dosage (adrenaline and noradrenaline) and indexed systemic vascular resistance trends. **B** Cardiac index trend. *AC/VC* volume-controlled assisted modality, *AC/PC* pressure-controlled assisted modality, *APRV* airway positive pressure release ventilation, *Pes* esophageal pressure

The decision to use the APRV modality was related to an improvement in cardiac function, a rise in the cardiac index to above 4.1 L/minute/m^2^ even after suspending dobutamine infusion (Fig. 5B), and hemodynamic improvement. This allowed for the suspension of continuous noradrenaline infusion, while levels of indexed systemic vascular resistance were kept between 1070 and 1860 dinas-seg-m^2^/cm^5^ (Fig. 5A). The indexed intrathoracic blood volume during APRV varied between 999 and 1335 ml/m^2^.

**Fig. 6:**
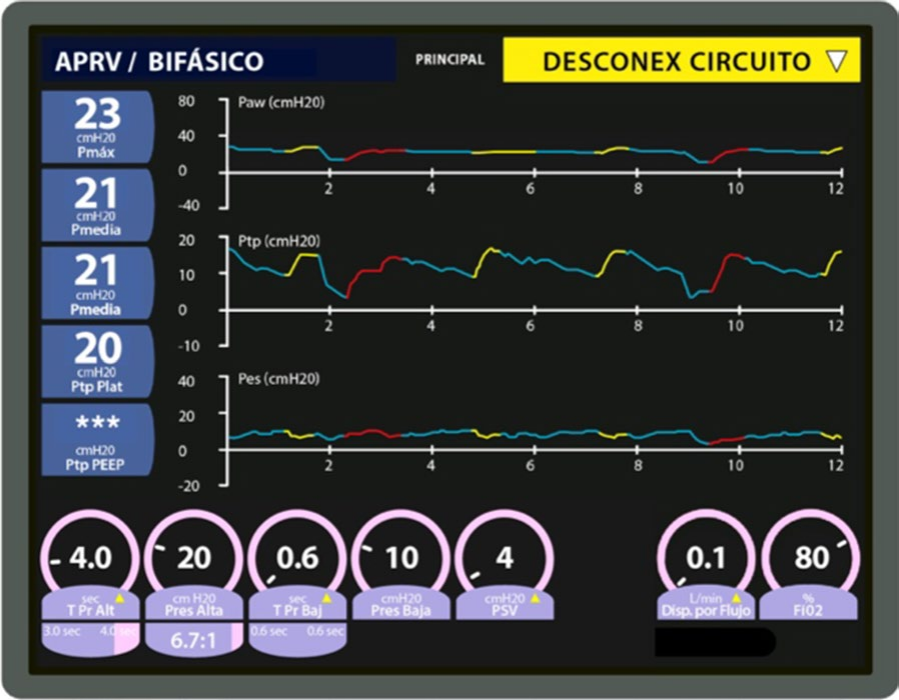
Mechanical ventilation programming during day 2 of esophageal balloon use. Airway positive pressure release ventilation modality with esophageal balloon

As described above, during controlled ventilation in parallel with hemodynamic complications, it was not possible to titrate the necessary PEEP to maintain transpulmonary pressure above 0 cmH_2_O (Fig. 3). However, the use of APRV was associated with reduced hemodynamic complications (Fig. 5) and kept the PLend-exp between 0 and 20 cmH_2_O despite a sustained PEEP high of 20 cmH_2_O (Fig. 6).

**Fig. 7:**
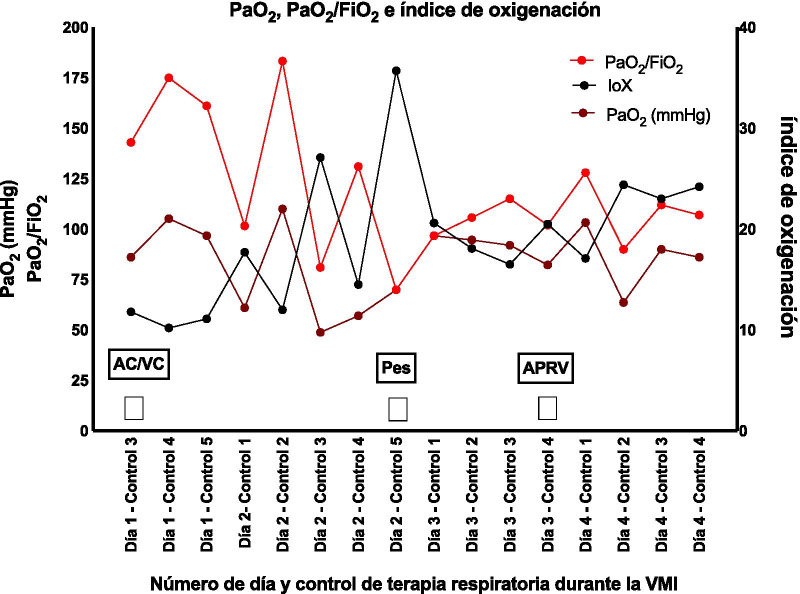
PaO_2_ trend, PaO_2_/FiO_2_ ratio, oxygenation index ventilatory modality. *AC/VC* volume-controlled assisted modality, *AC/PC* pressure-controlled assisted modality, *APRV* airway positive pressure release ventilation, *Pes* esophageal pressure

Following the start of Pes monitoring and APRV, the patient’s PaO_2_, PaO_2_/FiO_2_ ratio, and oxygenation index all varied less than they had under the controlled modality (Fig. 7). The P-high was adjusted to 2 cmH_2_O over plateau pressure and was adjusted according to the ventilatory graph, keeping transpulmonary pressures under 15 cmH_2_O. The P-low was adjusted according to the ventilatory graph, always maintaining transpulmonary pressures above 2 cmH_2_O. Pressure support was titrated to keep the spontaneous volume mobilized on the P-high close to 20% of its release volume, using the esophageal pressure graph to verify that it did not rise above 20 cmH_2_O of transpulmonary pressure.

## Weaning

On the seventh day of mechanical ventilation, the patient was in the appropriate condition to initiate supported weaning (CPAP with added support pressure), using 11 cmH_2_O mean airway pressure (Table 4, day 6). Under these conditions, a low-support spontaneous ventilation test was conducted, showing a rapid shallow breathing index (RSBI) of 34 and 57, a negative inspiratory force (NIF) of –27 cmH_2_O, and airway occlusion pressure in the first 100 milliseconds (P0.1) of –2 cmH_2_O, alongside the application of a negative cuff leak test. Considering these indicators, the patient was extubated with no complications, and weaning was consolidated with the support of a high-flow nasal cannula. Once weaning was consolidated, the patient was taken to the intermediary care unit.

**Table 4:** Respiratory therapy record—conventional mechanical ventilation with esophageal balloon and weaning

Day / round number	Day 5 / 1	Day 5 / 1	Day 5 / 3	Day 6 / 1	Day 6 / 2	Day 6 / 3	Day 7 / 1	Day 7 / 2	Day 7 / 3	Day 7 / 4
Mechanical ventilator modality	AC/PC	AC/PC	AC/PC	AC/PC	CPAP/PSV	CPAP/PSV	CPAP/PSV	CPAP/PSV	CPAP/PSV	CPAP/PSV
Programmed respiratory frequency (revolutions per minute)	16	15	15	15	–	–	–	–	–	–
Total respiratory frequency (revolutions per minute)	16	15	15	15	18	18	16	23	28	24
Programmed tidal volume (cmH_2_O)	12	12	12	12	–	–	–	–	–	–
Expiratory tidal volume (ml)	440	440	430	430	546	560	520	430	390	380
Expiratory minute volume (liters per minute)	6.9	6.7	6.3	6.3	8.2	10.6	8.9	10.1	8.7	9
Inspiratory time (seconds)	0.9	0.9	0.9	0.9	–	–	–	–	–	–
Expiratory time (seconds)	0.64	0.9	0.9	0.9	0.9	–	–	–	–	–
Maximum pressure (cmH_2_O)	28	25	25	25	25	24	24	15	12	13
Plateau pressure (cmH_2_O)	22.5	22.8	24	24	–	–	–	–	–	–
Mean airway pressure (cmH_2_O)	16	16	16	16	17	17	17	11	8	8
PEEP / PEEP Low (APRV) (cmH_2_O)	12	12	12	12	12	12	14	8	5	5
Support pressure (cmH_2_O)	–	–	–	–	?	12	12	10	7	7
FiO_2_ (%)	35	35	35	35	35	35	40	35	30	30
SpO_2_ (%)	100	100	100	100	100	96	98	100	100	100
Respiratory system compliance (Static)	34	34	34	–	–	–	–	–	–	–
PLend-insp (cmH_2_O)	8	12	8	10						
Plend-exp (cmH_2_O)	0	1	0	3						
pH	7.47	–	7.41	7.42	7.42	7.48	7.49	7.47	–	–
PCO_2_ (mmHg)	32.4	–	28.9	35.5	37.1	29.9	29.8?	32.8	–	–
PO_2_ (mmHg)	82	–	76.7	75.2	79.6	57.9	74.2	98.9	–	–
HCO_3_ (mmol/L)	23.1	–	24.1	22.9	23.9?	22	22.2	23.3	–	–
B.E.	0.2	–	−0.3	−0.8	−0.2	−0.2	0	0.5	–	–
Sat O_2_ (%)	96.8	–	95.5	95.5	94.7?	92.4	96.1	97.9	–	–
PaO_2_/FiO_2_	234	–	219	214	206?	165	185.5	282.5	–	–
Iox	6.8	–	7.3	7.5	7.8	10.3	9.1	3.8	–	–

AC/PC: Pressure-controlled assisted modality; CPAP: Continuous positive airway pressure; PSV: support pressure ventilation; PEEP: Positive end-expiratory pressure; FiO_2_: Fraction of inspired oxygen; PLend-insp: Transpulmonary pressure during end inspiration; PLend-exp: Transpulmonary pressure during end expiration; SpO_2_: Pulse oximetry saturation; PCO_2_: Arterial partial pressure of carbon dioxide; PO_2_: Arterial partial pressure of oxygen; HCO_3_: Bicarbonate; B.E: Base excess; SaO_2_: Arterial oxygen saturation; Iox: Oxygenation Index

## Discussion and conclusions

Although there is a general agreement on the reduction of tidal volume, plateau pressure, and driving pressure as key objectives of pulmonary care [2], the most adequate way to determine positive end-expiratory pressure is still debated, as it has been for decades [11].

There have been cases where healthy animals have suffered ventilator-induced lung injuries (VILI) when ventilation produces pulmonary overdistention [12]. Although it is rare to reach such scores in clinical practice, note that in ARDS a substantial region of the lung is heterogeneous, inducing stress and involving the doubling of locally applied pressure as a possible side effect [1]. Although this is noteworthy and requires consideration when titrating mechanical ventilation, beneficial effects have been reported in several clinical studies when sufficient PEEP was used to drive transpulmonary pressure at the end of the expiration from a negative (indicating the closure or collapse of expiration) to a positive transition (indicating sustained permeability in this zone) [13]. In the present case, PEEP titration aimed to achieve these objectives (Table 3, day 2, fifth round).

In other cases, the use of an esophageal balloon has been criticized since it does not directly measure pleural pressure in distant pulmonary regions. Yet results from a recent study indicate that esophageal pressure is reliably close to absolute pleural pressure throughout its isogravitational plane [14]. In addition to the above, the restrictive component of the thoracic cage is increased by the weight of abdominal and thoracic adipose tissue (BMI 29), exposing the need to investigate the contribution of thoracic elasticity to lung mechanics.

On the third day of mechanical ventilation, the patient was transferred to APRV, due to the potential benefits shown in a randomized clinical trial [15] and based on empirical results at our center. This modality maintains spontaneous respiration, and esophageal pressure screening in SDRA patients can be relevant, since deficient or excessive spontaneous force levels can lead to lesions on the lungs and diaphragm [16]. Secondly, calculated transpulmonary pressure is a useful indicator for clinicians to spot excessive spontaneous force-induced damage [17], which can worsen lesions [18]. Furthermore, when spontaneous effort toward the end of the inspiration takes place with considerable muscle relaxation, transpulmonary pressure can reveal transalveolar pressure (namely, the component for alveolar expansion) [7]. Figure 4 shows indicators for transpulmonary pressure below 20 cmH_2_O in APRV, which means that alveolar distention is even lower, despite maintaining a PEEP high of 20 cmH_2_O.

A recent case report on the usefulness of esophageal manometry during APRV implementation concludes that if transpulmonary pressure during release is unknown, then programming is being done blindly without knowing the frequency of alveolar collapse, as the report shows a transpulmonary pressure drop to below 0 cmH_2_O during expiration [19]. In our study, the use of APRV did not show transpulmonary pressure drops below 0 cmH_2_O, maintaining the pulmonary recruitment targets with esophageal manometry (Fig. 4). This also allowed the team to maintain stable oxygenation targets without considerable alterations, as had occurred prior to the use of esophageal manometry, and achieve even greater stability during APRV (Fig. 7).

APRV requires spontaneous respiratory cycles and, as such, is associated with lower sedation levels. In this study, APRV use required the suspension of continuous midazolam infusion, raising the RASS from −5 to −3, while maintaining the administration of dexmedetomidine and fentanyl (Fig. 5). The reduction in sedation was related to improvements in the hemodynamic profile parameters, likely due to an adrenergic rise and the spontaneous cycles that lead to intrathoracic pressure variation. The latter could cause increased venous return and preload, which can manifest itself in indexed intrathoracic blood volume from 999 to 1335 ml/m^2^. This allowed for the suspension of continuous noradrenaline infusion while maintaining indexed systemic vascular resistance scores between 1070 and 1860 dyne seconds/m^2^/cm^5^ (Fig. 6A) and cardiac index scores above 4.1 L/minute/m^2^ even after discontinuing dobutamine (Fig. 6B). These results are in line with the benefits described in the available literature on APRV use [6].

Asynchrony can worsen pulmonary lesions, as is the case in a “double trigger” event, where two consecutive inspirations are taken after a single respiratory effort [20], thus doubling supplied tidal volume (TV). Double trigger cases are more frequent in patients with a greater respiratory impulse [21]. The adverse impact of asynchrony in patients on a ventilator is becoming more widely recognized, and the literature suggests a link between the rise of asynchrony and mortality [22]. Conventional pressure and flow monitoring over time can hide much of the interaction between patients and respirators, but esophageal pressure data can help detect asynchrony more easily [7]. Hence, careful monitoring of patient/ventilator interaction can help determine mechanical ventilation programming and sedoanalgesia levels [7]. Figure 4 shows that all the patient’s spontaneous efforts (negative deflection in the transpulmonary and esophageal pressure curve) are accompanied by supported ventilation or APRV mandatory cycles.

There is a growing need for research on APRV configuration considering its increased use. Since APRV involves spontaneous cycles, it allows for reduced sedation levels, improving adrenergic activity and diminishing vasoactive drug requirements (Figs. 5, 6). This report shows the usefulness of mechanical ventilation programming for lung protection using transpulmonary pressure monitoring. The report also suggests the usefulness of invasive mechanical ventilation Pes monitoring, both in controlled modalities and those allowing spontaneous respiration, such as APRV.

It is important to note that the patient’s overall medical condition, hemodynamic compromise, and the consequent fluctuating sedation levels did not allow the team to maintain a single ventilating modality and a steady programming, which in turn would have allowed for a more complete temporal assessment and description of Pes monitoring. Additionally, there was no continuous quantification of esophageal catheterization indicators in modalities that allow spontaneous respiration, and they were merely monitored. Their quantification can be established by directly measuring the Pes and Ptp pressure/time curve. However, these data were not recorded, and as such, the study only exemplifies their usefulness in APRV.

This report describes Pes monitoring in different modalities. Further studies are required to better understand its use in patients who require the titration of their mechanical ventilation programming, both in controlled modalities and those allowing spontaneous respiration. However, it is necessary to perform introduction, calibration, and corroboration techniques to adequately position the esophageal balloon, both through gentle external manual epigastric compression [1], and occlusion or Baydur tests [10].

## Data Availability

Not applicable.

## Abbreviations

ABGs: Arterial blood gas test
AC/VC: Volume-controlled assisted modality
AC/PC: Pressure-controlled assisted modality
APACHE II: Acute physiology and chronic health disease classification system II
APRV: Airway pressure release ventilation
ARDS: Acute respiratory distress syndrome
ARDSNet: Acute respiratory distress syndrome network
B.E.: Base excess
BPM: Breaths per minute
CPAP: Continuous positive airway pressure
FiO_2_: Fraction of inspired oxygen
ICU: Intensive care unit
MAP: Mean arterial pressure
NIF: Negative inspiratory force
P.01.: Airway occlusion pressure in the first 100 milliseconds
PEEP: Positive end-expiratory pressure
Pes: Esophageal pressure
PLend-insp: Transpulmonary pressure during end inspiration
PLend-exp: Transpulmonary pressure during end expiration
Ptp: Transpulmonary pressure
RASS: Richmond Agitation–Sedation Scale
RSBI: Rapid shallow breathing index
SpO_2_: Pulse oximetry saturation
SaO_2_: Arterial oxygen saturation
VILI: Ventilator-induced lung injuries
VT: Tidal volume

## References

[1] Akoumianaki E, Maggiore SM, Valenza F, Bellani G, Jubran A, Loring SH, Pelosi P, Talmor D, Grasso S, Chiumello D, Guérin C, Patroniti N, Ranieri VM, Gattinoni L, Nava S, Terragni PP, Pesenti A, Tobin M, Mancebo J, Brochard L, PLUG Working Group (Acute Respiratory Failure Section of the European Society of Intensive Care Medicine) (2016). Esophageal and transpulmonary pressure in the clinical setting: meaning, usefulness and perspectives. Intensive Care Med.

[2] Definition Task Force ARDS, Ranieri VM, Rubenfeld GD, Thompson BT, Ferguson ND, Caldwell E, Fan E, Camporota L, Slutsky AS (2012). Acute respiratory distress syndrome: the Berlin Definition. JAMA.

[3] Downs JB, Stock MC (1987). Airway pressure release ventilation: a new concept in ventilatory support. Crit Care Med.

[4] Daoud EG, Farag HL, Chatburn RL (2012). Airway pressure release ventilation: what do we know?. Respir Care.

[5] Hirani A, Marik PE, Plante LA. Airway pressure-release ventilation in pregnant patients with acute respiratory distress syndrome: a novel strategy. Respir Care. 2009; 54(10): 1405–1408. http://rc.rcjournal.com/content/54/10/1405. 19796422

[6] Facchin F, Fan E (2015). Airway pressure release ventilation and high-frequency oscillatory ventilation: potential strategies to treat severe hypoxemia and prevent ventilator-induced lung injury. Respir Care.

[7] Yoshida T, Brochard L (2018). Ten tips to facilitate understanding and clinical use of esophageal pressure manometry. Intensive Care Med.

[8] Protti A, Cressoni M, Santini A (2011). Lung stress and strain during mechanical ventilation: any safe threshold?. Am J Respir Crit Care Med.

[9] Niknam J, Chandra A, Adams AB, Nahum A, Ravenscraft SA, Marini JJ (1994). Effect of a nasogastric tube on esophageal pressure measurement in normal adults. Chest.

[10] Baydur A, Behrakis PK, Zin WA, Jaeger M, Milic-Emili J (1982). A simple method for assessing the validity of the esophageal balloon technique. Am Rev Respir Dis.

[11] Marini JJ (2018). Should we titrate positive end-expiratory pressure based on an end-expiratory transpulmonary pressure?. Ann Transl Med..

[12] Cressoni M, Cadringher P, Chiurazzi C, Amini M, Gallazzi E, Marino A, Brioni M, Carlesso E, Chiumello D, Quintel M, Bugedo G, Gattinoni L (2014). Lung inhomogeneity in patients with acute respiratory distress syndrome. Am J Respir Crit Care Med.

[13] Mead J, Takishima T, Leith D (1970). Stress distribution in lungs: a model of pulmonary elasticity. J Appl Physiol.

[14] Crotti S, Mascheroni D, Caironi P (2001). Recruitment and derecruitment during acute respiratory failure: a clinical study. Am J Respir Crit Care Med.

[15] Zhou Y, Jin X, Lv Y, Wang P, Yang Y, Liang G, Wang B, Kang Y (2017). Early application of airway pressure release ventilation may reduce the duration of mechanical ventilation in acute respiratory distress syndrome. Intensive Care Med.

[16] Goligher EC, Fan E, Herridge MS, Murray A, Vorona S, Brace D, Rittayamai N, Lanys A, Tomlinson G, Singh JM, Bolz SS, Rubenfeld GD, Kavanagh BP, Brochard LJ, Ferguson ND (2015). Evolution of diaphragm thickness during mechanical ventilation. Impact of inspiratory effort. Am J Respir Crit Care Med.

[17] Mauri T, Langer T, Zanella A, Grasselli G, Pesenti A (2016). Extremely high transpulmonary pressure in a spontaneously breathing patient with early severe ARDS on ECMO. Intensive Care Med.

[18] Yoshida T, Uchiyama A, Matsuura N, Mashimo T, Fujino Y (2013). The comparison of spontaneous breathing and muscle paralysis in two different severities of experimental lung injury. Crit Care Med.

[19] Daoud EG, Yamasaki KH, Nakamoto K, Wheatley D (2018). Esophageal pressure balloon and transpulmonary pressure monitoring in airway pressure release ventilation: a different approach. Can J Respir Therapy CJRT (Revue Canadienne de la Therapie Respiratoire RCTR).

[20] Pohlman MC, McCallister KE, Schweickert WD (2008). Excessive tidal volume from breath stacking during lung-protective ventilation for acute lung injury. Crit Care Med.

[21] Thille AW, Rodriguez P, Cabello B, Lellouche F, Brochard L (2006). Patient-ventilator asynchrony during assisted mechanical ventilation. Intensive Care Med.

[22] Blanch L, Villagra A, Sales B (2015). Asynchronies during mechanical ventilation are associated with mortality. Intensive Care Med.

[23] Gattinoni L, Pelosi P, Suter PM (1998). Acute respiratory distress syndrome caused by pulmonary and extrapulmonary disease: different syndromes?. Am J Respir Crit Care Med.

